# Case Report: Three rare cases of pseudomyogenic hemangioendothelioma with atypical skeletal involvement

**DOI:** 10.3389/fmed.2026.1934676

**Published:** 2026-09-29

**Authors:** Meizheng Dang, Buyi Zhang, Wen Xu, Pintong Huang

**Affiliations:** 1 Department of Ultrasound in Medicine, The Second Affiliated Hospital of Zhejiang University School of Medicine, Hangzhou, Zhejiang, China; 2 Department of Pathology, The Second Affiliated Hospital of Zhejiang University School of Medicine, Hangzhou, Zhejiang, China

**Keywords:** case report, computed tomography, histopathological examination, immunohistochemical, magnetic resonance imaging, positron emission tomography/computed tomography, pseudomyogenic hemangioendothelioma

## Abstract

**Introduction:**

Pseudomyogenic hemangioendothelioma (PHE) is a rare vascular neoplasm of intermediate malignant potential that predominantly affects young adult men and most frequently arises in the lower extremities. Involvement of unusual skeletal sites is uncommon, and the nonspecific imaging features of PHE may contribute to diagnostic uncertainty.

**Case report:**

We report three cases of PHE involving unusual osseous locations, including the thoracic spine, femur, and humerus, with one case showing concomitant soft tissue involvement of the upper arm. Multimodal imaging, including computed tomography (CT), magnetic resonance imaging (MRI), and positron emission tomography/computed tomography (PET/CT), demonstrated osteolytic bone destruction, soft tissue extension, heterogeneous enhancement, and increased metabolic activity. All patients underwent individualized surgical management according to tumor location and disease extent. The final diagnosis was established by histopathological and immunohistochemical (IHC) evaluation, particularly FOSB positivity and endothelial marker expression.

**Conclusion:**

PHE should be considered in the differential diagnosis of atypical bone and soft tissue lesions, even when they occur at uncommon anatomic sites or in patients outside the typical demographic profile. Comprehensive imaging assessment, histopathological confirmation, individualized treatment, and long-term follow-up are essential for accurate diagnosis and optimal clinical management.

## Introduction

Pseudomyogenic hemangioendothelioma (PHE), also known as epithelioid sarcoma-like hemangioendothelioma, is a rare vascular neoplasm of intermediate malignant potential that usually manifests as painless or mildly tender nodules or masses and may present with multifocal disease (1). PHE predominantly affects young adult men and most frequently arises in the extremities, particularly the lower limbs (2–4). Although PHE typically involves the skin and soft tissues, multifocal disease may extend across multiple anatomic planes, including the dermis, subcutaneous tissue, skeletal muscle, and bone, thereby increasing diagnostic complexity (3, 5).

Diagnosing PHE remains challenging because its imaging findings are generally nonspecific and multifocal lesions may be present. Computed tomography (CT), magnetic resonance imaging (MRI), and positron emission tomography/computed tomography (PET/CT) are useful for assessing lesion extent, skeletal involvement, and metabolic activity, but they cannot replace histopathological confirmation (6–8). Furthermore, despite its generally indolent clinical course, PHE has a considerable propensity for local recurrence, underscoring the need for long-term follow-up (3, 4).

Herein, we report three cases of PHE involving atypical skeletal sites, including the thoracic spine, femur, and humerus, with one case showing associated soft tissue involvement in the upper arm. This case series aims to increase awareness of this rare tumor in unusual osseous and soft tissue locations and highlights the importance of comprehensive imaging assessment combined with histopathological analysis for accurate diagnosis.

## Case report 1

A 61-year-old woman presented to our outpatient clinic with a 2-month history of persistent back pain without an obvious precipitating factor. She described the pain as a pulling sensation radiating from the back to the sternum, which had progressively worsened and interfered with sleep.

Physical examination revealed localized tenderness over the mid-thoracic spine, approximately at the sixth to seventh thoracic vertebral levels (T6–T7). Thoracic radiography demonstrated decreased density of the T7 vertebral body with concomitant degenerative changes (Figure 1A). Contrast-enhanced CT of the thoracic spine revealed osteolytic destruction of the T7 vertebral body and posterior elements, accompanied by an associated soft tissue mass (Figure 1B). The lesion extended to the adjacent T6 and T8 vertebral bodies and protruded into the spinal canal (Figure 1C). Subsequent contrast-enhanced MRI further demonstrated a destructive lesion centered in the T7 vertebral body, with soft tissue extension, secondary spinal canal stenosis, and spinal cord compression. The lesion showed marked heterogeneous enhancement (Figures 1D,E). Based on these imaging findings, a malignant primary spinal tumor or metastatic disease was suspected. After admission, whole-body PET/CT revealed intense radiotracer uptake in the T7 vertebral lesion and adjacent involved structures, with a maximum standardized uptake value (SUVmax) of 23.87. No additional hypermetabolic lesions were detected elsewhere (Figure 1F).

**Figure 1:**
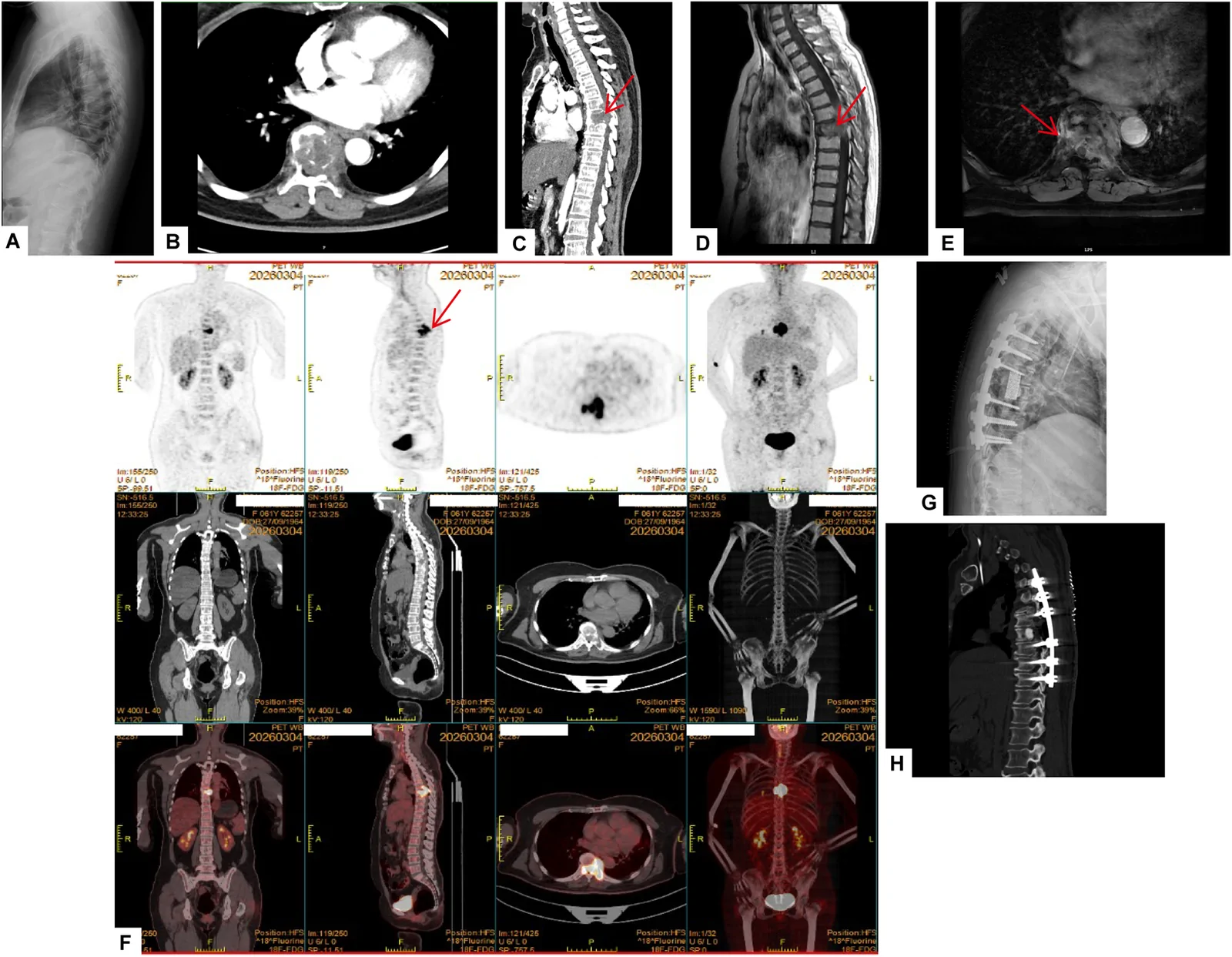
Imaging findings of thoracic vertebral PHE. **(A)** Thoracic radiography showed decreased density of the T7 vertebral body. **(B,C)** Contrast-enhanced CT revealed osteolytic destruction of T7 with adjacent soft tissue extension and spinal canal involvement (arrow). **(D,E)** Contrast-enhanced MRI showed spinal canal stenosis and cord compression, with marked heterogeneous enhancement of the lesion (arrow). **(F)** PET/CT showed intense radiotracer uptake in the thoracic vertebral lesion (arrow). **(G,H)** Postoperative radiography and CT showed satisfactory spinal reconstruction and internal fixation.

After multidisciplinary discussion and written informed consent had been obtained from the patient and her family, surgical resection of the thoracic vertebral lesion was performed. The procedure consisted of tumor resection, thoracic laminectomy and decompression, posterior thoracic spinal fusion with pedicle screw fixation, titanium mesh reconstruction, and bone cement augmentation. The operation was completed successfully, and the postoperative course was uneventful. Final histopathological examination showed diffusely and patchily arranged tumor cells infiltrating the surrounding adipose tissue (Supplementary Figure S1A). Focal tumor necrosis was observed in some areas, accompanied by small foci of trabecular bone formation, collagen deposition, scattered lymphocytic infiltration, and hemosiderin deposition (Supplementary Figures S1B–D). The tumor cells ranged from short spindle-shaped to epithelioid and had indistinct cell borders. Their nuclei were vesicular, with small nucleoli, fine chromatin, and inconspicuous mitotic activity (Supplementary Figure S1E). Some tumor cells exhibited abundant eosinophilic cytoplasm and eccentrically displaced nuclei, showing rhabdomyoblast-like morphology (Supplementary Figure S1F). Further immunohistochemical (IHC) analysis showed partial positivity for CK-PAN, positivity for CD31, FOSB, ERG and INI1/SMARCB1 (Supplementary Figures S1G–K), whereas CD34 and CAMTA1 were negative in tumor cells (Supplementary Figures S1L,M). The tumor cells were also negative for desmin, supporting endothelial differentiation rather than true skeletal muscle differentiation (Supplementary Figure S1N). The Ki-67 proliferation index was approximately 10%, reaching 20% in hot spots, and p53 showed partial positivity, indicating a relatively low-grade malignant potential. Collectively, these histopathological and IHC findings helped exclude neurogenic tumors, solitary fibrous tumor, synovial sarcoma, and epithelioid sarcoma, thereby supporting the diagnosis of PHE.

Postoperative thoracic radiography and CT demonstrated satisfactory alignment and reconstruction of the operated spinal segment after resection of the thoracic lesion. The internal fixation system, titanium mesh, artificial vertebral body, and bone cement were appropriately positioned (Figures 1G,H). No immediate postoperative complications were observed. After discharge, the patient was advised to undergo regular follow-up to assess spinal stability and monitor for local recurrence. At 3- and 6-month postoperative follow-up, no clinical evidence of local recurrence was observed; however, follow-up imaging was unavailable for review.

## Case report 2

A 36-year-old woman presented to our hospital with a 1-month history of pain and restricted mobility in the left lower limb after squatting. Approximately 18 months earlier, she had undergone resection of a left femoral tumor at another institution, where the lesion was interpreted as a low- to intermediate-grade malignant neoplasm. IHC staining demonstrated focal positivity for CK-PAN and RB1; positivity for SATB2, H3K27me3, and FOSB; scattered ERG positivity; weak CD31 expression; and negativity for desmin, p53, and caldesmon. Molecular pathological analysis using fluorescence *in situ* hybridization (FISH) showed no MDM2 gene amplification. Based on the available histopathological and IHC findings, PHE was favored; however, osteosarcoma could not be entirely excluded because the complete original pathology report was unavailable.

Physical examination revealed a well-healed postoperative scar on the left thigh, measuring approximately 30 cm in length. Radiography of the left femur demonstrated postoperative changes, with the internal fixation device remaining *in situ* (Figure 2A). CT further showed postoperative changes involving the proximal to mid femur, with heterogeneous bone density, partial osseous defects, and a localized soft tissue mass (Figure 2B). Subsequent MRI demonstrated abnormal marrow signal intensity and morphological distortion of the proximal to mid femur, with mild swelling of the surrounding soft tissues (Figure 2C). These findings were suspicious for tumor recurrence. After admission, PET/CT demonstrated postoperative changes in the proximal left femur and an expansile soft tissue mass surrounding the proximal femur, measuring approximately 13.26 cm in maximum diameter. The lesion was accompanied by localized bone destruction and markedly increased radiotracer uptake, with an SUVmax of 32.92 (Figure 2D). Based on the imaging findings and previous pathological results, recurrence of a low- to intermediate-grade malignant lesion was suspected, with PHE favored as the most likely diagnosis.

**Figure 2:**
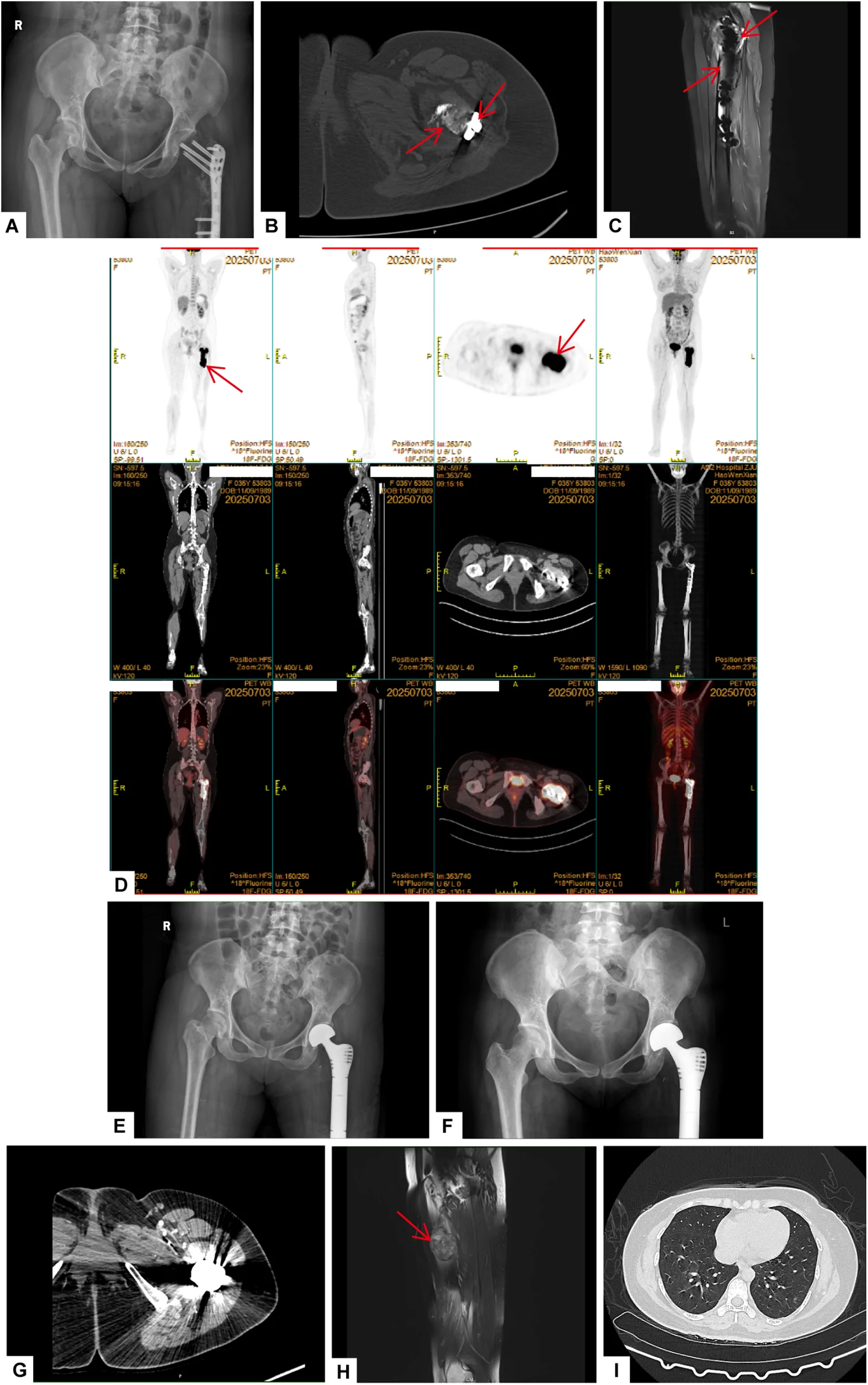
Imaging findings of femoral PHE and postoperative follow-up. **(A)** Radiography showed postoperative changes of the left femur with the internal fixation device *in situ*. **(B)** CT demonstrated osseous destruction and an associated soft tissue component (arrows). **(C)** MRI revealed abnormal marrow signal and surrounding soft tissue changes (arrows). **(D)** PET/CT showed marked radiotracer uptake around the proximal femoral lesion (arrows). **(E,F)** Postoperative radiographs showed satisfactory positioning of the femoral prosthesis. **(G)** Eight months after surgery, contrast-enhanced CT showed an enhancing soft tissue mass in the medial thigh. **(H)** MRI demonstrated a heterogeneous soft tissue lesion with heterogeneous hyperintensity on T2WI (arrow). **(I)** Chest CT showed multiple bilateral pulmonary nodules.

After discussion with the patient and her family, resection of the left femoral lesion combined with artificial femoral head replacement was performed. The procedure was uneventful. Postoperative histopathological examination showed tumor cells were arranged in sheets and infiltrated the adipose tissue. No tumor necrosis was identified. Foci of trabecular bone formation were observed (Supplementary Figures S2A,B), accompanied by scattered lymphocytic and neutrophilic infiltration (Supplementary Figures S2C,D). Clusters of multinucleated giant cells were present in some areas. Some tumor cells exhibited abundant eosinophilic cytoplasm and eccentrically displaced nuclei, resembling rhabdoid morphology (Supplementary Figures S2E,F). Further IHC analysis showed positivity for CK-PAN, CD31, FLI1, ERG, FOSB and INI1/SMARCB1 (Supplementary Figures S2G–L), while CD34 and CAMTA1 were negative in tumor cells (Supplementary Figures S2M,N). The Ki-67 proliferation index was approximately 10%. Taken together, these histopathological and IHC findings supported the diagnosis of PHE. Both postoperative and follow-up imaging demonstrated that the femoral prosthesis was well positioned, with satisfactory alignment and adequate bone cement distribution around the prosthesis (Figures 2E,F).

Eight months after surgery, the patient noticed a palpable mass in the left thigh and returned to our hospital for further evaluation. Contrast-enhanced CT of the left femur revealed a soft tissue mass measuring approximately 47 × 59 mm in the upper-to-middle medial thigh. The lesion showed relatively well-defined margins, a lobulated contour, and marked heterogeneous enhancement, with multiple intralesional vessels (Figure 2G). MRI also demonstrated a heterogeneous soft-tissue mass in the same region, showing heterogeneous hyperintensity on T2-weighted images (T2WI) (Figure 2H). These imaging findings were suggestive of local tumor recurrence. Subsequent chest CT revealed multiple bilateral pulmonary nodules, some of which were considered suspicious for metastasis given the patient's clinical history (Figure 2I). A CT-guided fine-needle aspiration biopsy of the thigh mass was subsequently performed. Pathological examination showed that the tumor was composed of epithelioid and spindle cells with cellular atypia, consistent with a low-grade malignant neoplasm (Supplementary Figures S3A,B). IHC demonstrated positivity for FOSB, ERG, focal positivity for CD31, and CD34 expression restricted to vascular structures; the Ki-67 proliferation index was approximately 10%. Collectively, these findings were consistent with recurrent PHE. Given the risk of further disease progression and the potential need for repeat surgery, the patient's condition and treatment options were discussed with her and her family. The patient elected to be discharged and continue treatment at a local hospital. After discharge, the patient received systemic chemotherapy at a local hospital. No biopsy of the pulmonary nodules was performed; therefore, whether they represented metastases could not be histologically determined. At the 3-month follow-up after treatment initiation, the patient was alive and clinically stable, and follow-up imaging reportedly showed no apparent progression of either the recurrent thigh lesion or the bilateral pulmonary nodules. However, the corresponding follow-up imaging studies were unavailable for review.

## Case report 3

A 20-year-old man presented to our hospital with a mass in the right upper arm that had been present for more than 4 months. He reported no limb swelling, numbness, weakness, or other associated discomfort. The patient initially received conservative treatment at a local hospital; however, the mass gradually enlarged over the subsequent months, and mild pain developed approximately 2 months before admission. CT performed at the local hospital revealed a ground-glass lesion within the medullary cavity of the proximal right humerus, raising the possibility of fibrous dysplasia. MRI suggested a subcutaneous hemangioma in the right upper arm and abnormal signal intensity within the medullary cavity of the proximal humerus; however, the detailed radiological report was unavailable.

Physical examination revealed a palpable mass in the right upper arm. Plain radiography and CT of the right humerus demonstrated heterogeneous bone density within the proximal humeral medullary cavity (Figures 3A,B). Contrast-enhanced MRI of the right upper arm showed multiple lesions in the proximal right humerus, the largest measuring approximately 14 × 16 × 27 mm (Figure 3C). The dominant lesion was located within the medullary cavity and had well-defined margins. It appeared mildly hypointense to isointense on T1-weighted images (T1WI) (Figure 3D) and mildly hyperintense on T2WI images (Figure 3E). In addition, multiple small nodules were identified in the subcutaneous tissue and intramuscular region of the proximal right upper arm, with the largest measuring approximately 11 × 5 × 11 mm. These nodules were mildly hypointense on T1WI (Figure 3F) and mildly hyperintense on T2WI (Figure 3G). Based on these imaging features, the lesions were initially considered more likely to represent benign vascular lesions, such as hemangiomas.

**Figure 3:**
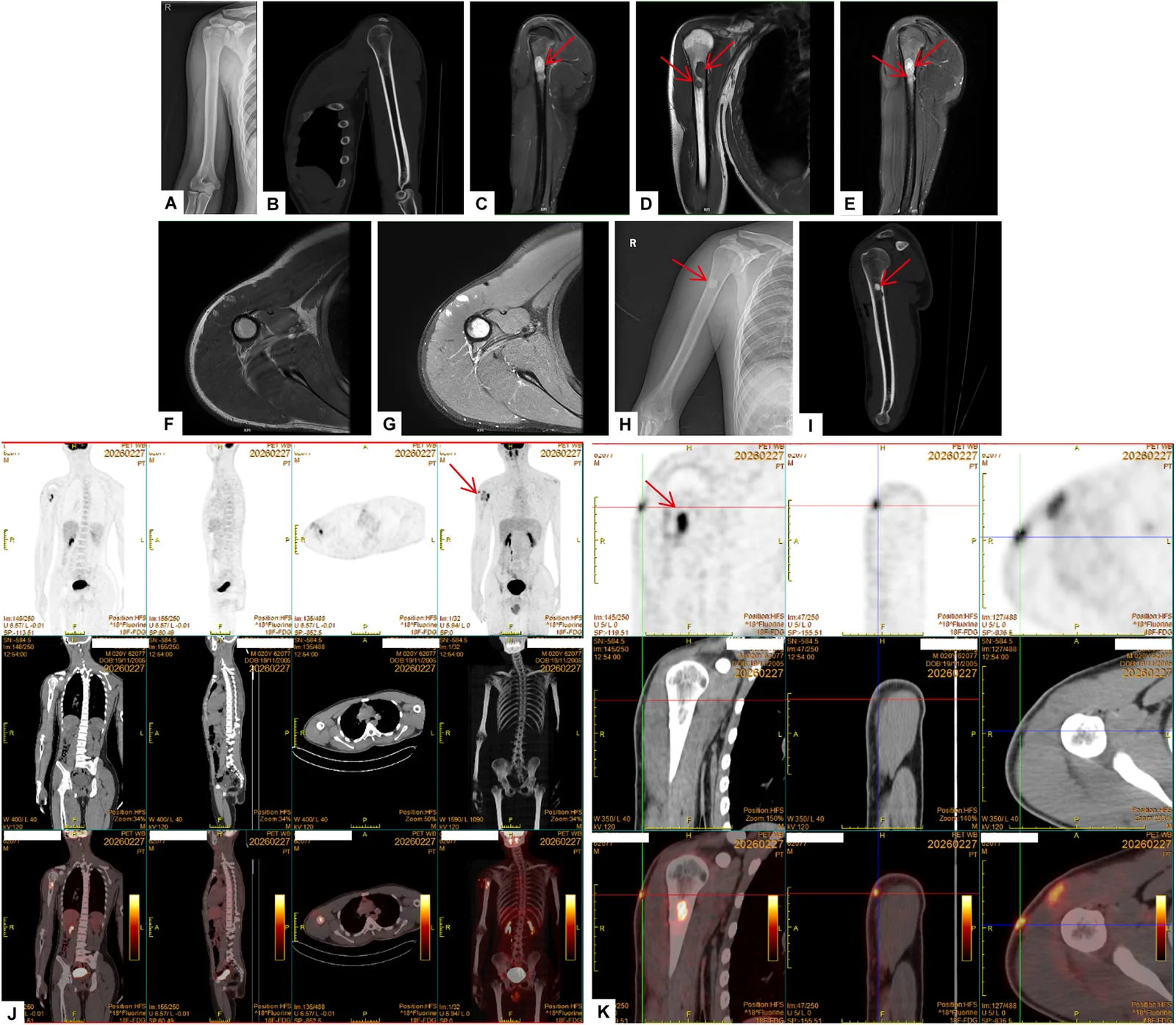
Imaging findings of humeral and upper-arm soft tissue PHE. **(A,B)** Radiography and CT showed heterogeneous density within the proximal humeral medullary cavity. **(C)** Contrast-enhanced MRI showed multiple lesions in the proximal right humerus (arrow). **(D)** The dominant intramedullary lesion was well defined and mildly hypointense to isointense on T1WI (arrows). **(E)** The lesion was mildly hyperintense on T2WI (arrows). **(F,G)** MRI showed multiple small subcutaneous and intramuscular nodules in the proximal right upper arm, with mild hypointensity on T1WI and mild hyperintensity on T2WI. **(H,I)** Postoperative radiography and CT showed bone cement deposition within the proximal humerus (arrow). **(J)** PET/CT showed increased radiotracer uptake adjacent to the surgical site in the proximal right humerus (arrow). **(K)** PET/CT showed increased radiotracer uptake in the subcutaneous and intramuscular nodules of the proximal right upper arm (arrow).

After surgical contraindications had been excluded and the treatment plan had been discussed in detail with the patient and his family, the patient underwent open biopsy of the right humeral lesion, bone cement filling of the osseous defect, and excision of the soft tissue lesion in the right upper arm. The operation was completed without intraoperative complications. Pathological examination showed that the tumor cells were arranged in sheets and infiltrated the skeletal muscle (Supplementary Figure S4A). Hemangioendothelioma-like slit-like spaces were observed, with minimal lymphocytic infiltration (Supplementary Figure S4B). Foci of trabecular bone formation were also present (Supplementary Figure S4C). The tumor cells had indistinct borders and ranged from epithelioid to spindle-shaped morphology (Supplementary Figure S4D). Due to frozen-section artifact, the tumor nuclei appeared hyperchromatic, with poorly defined chromatin and nucleoli, and mitotic figures were inconspicuous. In some areas, the tumor cells exhibited abundant eosinophilic cytoplasm and eccentrically displaced nuclei, showing rhabdomyoblast-like morphology (Supplementary Figure S4E). Further IHC analysis showed positivity for CK-PAN, FOSB, INI1/SMARCB1, focal positivity for CD31, and vascular positivity for CD34 (Supplementary Figures S4F–J). The tumor cells were negative for CD34, desmin, and CAMTA1 (Supplementary Figures S4K–L). The Ki-67 proliferation index was approximately 10%. Taken together, these histopathological and IHC findings supported the diagnosis of PHE.

Postoperative radiography and CT demonstrated changes related to biopsy and bone cement filling of the right humeral lesion. The medullary cavity of the proximal right humerus showed heterogeneous density with nodular high-density bone cement deposition, without obvious cortical destruction or periosteal reaction (Figures 3H,I). PET/CT demonstrated heterogeneous density and high-density filling material within the medullary cavity of the proximal right humerus. Increased radiotracer uptake was noted adjacent to the surgical region, with a SUVmax of 8.53 (Figure 3J). In addition, small nodules demonstrating increased radiotracer uptake were identified in the subcutaneous and intramuscular tissues of the proximal right upper arm, with an SUVmax of 5.06 (Figure 3K). No abnormal radiotracer distribution was detected in the remaining skeleton or bone marrow. At the 3- and 6-month postoperative follow-up visits, the patient showed no clinical evidence of local recurrence, although follow-up imaging was unavailable for review.

## Discussion

PHE is a rare vascular neoplasm of intermediate malignant potential that predominantly affects young men and most commonly arises in the extremities, but it may also occur at unusual skeletal sites and extend across multiple tissue planes (3, 4). Herein, we report three cases of PHE involving the thoracic spine, femur, and humerus, with one case showing concomitant soft tissue involvement in the upper arm. The clinical, radiological, and pathological characteristics of all three cases are summarized in Table 1. Notably, two patients were women, and one patient developed postoperative local recurrence and pulmonary nodules suspicious for metastasis. These cases suggest that PHE may present with atypical demographic and anatomic features, and that involvement of uncommon osseous sites, together with nonspecific imaging findings, may complicate preoperative diagnosis (4, 9).

**Table 1:** Clinical, radiological, and pathological characteristics of the three patients with PHE.

Case	Clinical characteristics	Radiological findings	Pathological findings
Case 1	**Age/sex:** 61 years/female **Presentation:** Persistent back pain for 2 months **Location:** T7 vertebra, with extension to T6 and T8	**CT:** Osteolytic vertebral destruction, associated soft tissue mass, and spinal canal involvement **MRI:** Heterogeneous enhancement, spinal canal stenosis, and spinal cord compression **PET/CT:** Increased uptake in the thoracic vertebral lesion; SUVmax 23.87	**Histology:** Spindle-to-epithelioid cells, rhabdomyoblast-like morphology, adipose tissue infiltration, and focal necrosis **IHC positive:** FOSB, CD31, ERG, INI1/SMARCB1, and CK-PAN (focal) **IHC negative:** CAMTA1, CD34 and desmin **Ki-67:** Approximately 10%; up to 20% in hot spots
Case 2	**Age/sex:** 36 years/female **Presentation:** Left lower-limb pain and restricted mobility for 1 month; previous femoral tumor resection **Location:** Left proximal-to-mid femur; medial thigh recurrence	**CT:** Femoral bone destruction and associated soft tissue mass; follow-up CT showed a recurrent medial thigh lesion and multiple bilateral pulmonary nodules **MRI:** Abnormal femoral marrow signal; follow-up MRI showed heterogeneous T2 hyperintensity in the recurrent medial thigh lesion. **PET/CT:** Increased uptake in the femoral lesion; SUVmax 32.92	**Histology:** Tumor cells arranged in sheets, rhabdoid morphology, adipose tissue infiltration, and multinucleated giant cells **IHC positive:** FOSB, CD31, ERG, FLI1, INI1/SMARCB1, and CK-PAN **IHC negative:** CAMTA1 and CD34 **Ki-67:** Approximately 10%
Case 3	**Age/sex:** 20 years/male **Presentation:** Right upper-arm mass for more than 4 months; subsequent mild pain **Location:** Right proximal humerus and adjacent upper-arm soft tissues	**CT:** Heterogeneous density within the proximal humeral medullary cavity **MRI:** Multifocal humeral and upper-arm lesions with mild T1 hypointensity and T2 hyperintensity **PET/CT:** Postoperative uptake in the humerus and upper-arm soft tissues; SUVmax 8.53 and 5.06, respectively	**Histology:** Spindle-to-epithelioid cells, rhabdomyoblast-like morphology, skeletal muscle infiltration, and slit-like vascular spaces **IHC positive:** FOSB, CK-PAN, INI1/SMARCB1, and CD31 (focal) **IHC negative:** CAMTA1 and desmin; CD34 negative in tumor cells with vascular positivity **Ki-67:** Approximately 10%

PHE, pseudomyogenic hemangioendothelioma; CT, computed tomography; MRI, magnetic resonance imaging; PET/CT, positron emission tomography/computed tomography; SUVmax, maximum standardized uptake value; IHC, immunohistochemical.

The pathogenesis of PHE remains incompletely understood; however, recurrent rearrangements involving FOSB-related genes are currently regarded as a key molecular event in this tumor (10–12). The SERPINE1-FOSB fusion is the most common alteration and results in transcriptional activation and nuclear overexpression of FOSB, providing important mechanistic and diagnostic implications (11). Other FOSB fusion partners, including ACTB, WWTR1, and CLTC, have also been reported (10, 12, 13). Although large clinical series have shown a clear male predominance, PHE has also been increasingly recognized in women among reported cases (3, 4, 14). At present, there is no conclusive evidence that PHE in women is driven by distinct hormone-dependent mechanisms. Instead, available molecular studies suggest that FOSB-related alterations, rather than sex-specific factors, play a central role in tumorigenesis (10–12). Accordingly, the occurrence of PHE in the two female patients in the present case series remains within the recognized disease spectrum, particularly because the histological morphology, endothelial marker expression, and FOSB positivity supported the diagnosis. Another notable feature of this case series is that all three patients showed skeletal involvement, affecting the thoracic vertebra, femur, and humerus. This finding may reflect the propensity of PHE to extend across multiple anatomic planes, including the subcutaneous tissue, skeletal muscle, and bone (3, 5, 15, 16). Moreover, the increasing number of reported primary or bone-predominant PHE cases further supports skeletal involvement as an important component of the clinicopathological spectrum of this rare tumor (15, 16). Collectively, the female patients and extensive skeletal involvement described in this case series highlight the broader clinical spectrum of PHE and underscore the need to consider this diagnosis even in patients with atypical demographic features or unusual anatomic locations.

Radiologically, PHE lacks specific imaging features and may mimic benign bone lesions, primary malignant bone tumors, metastatic disease, or vascular malformations (8, 9, 15). In skeletal lesions, radiography and CT may show well-defined osteolytic lesions or aggressive bone destruction, whereas MRI is useful for evaluating bone marrow involvement, soft tissue extension, and multifocal disease (9, 15, 16). These nonspecific features make the differential diagnosis of PHE quite challenging; particularly when PHE occurs in the spine or long bones, the lesions may be misdiagnosed as fibrous dysplasia, giant cell tumor, osteosarcoma, metastatic tumors, or other vascular tumors (8, 9, 15). PET/CT can demonstrate increased metabolic activity and help identify multifocal osseous and soft tissue lesions; however, increased FDG uptake is not specific to PHE and therefore cannot replace histopathological confirmation (7, 8, 16). In the present cases, CT, MRI, and PET/CT were valuable for delineating lesion extent, assessing skeletal and soft tissue involvement, and detecting multifocal disease. Nevertheless, a definitive diagnosis of PHE required an integrated assessment of imaging findings, histopathology, IHC, and, when necessary, molecular testing.

Histologically, PHE is typically composed of spindle-shaped to epithelioid tumor cells with abundant eosinophilic cytoplasm, and these morphological features may overlap with those of epithelioid sarcoma, myogenic tumors, fibrous lesions, osteosarcoma, and metastatic carcinoma (2, 5, 15). IHC is essential for diagnosis because PHE usually shows evidence of both epithelial and endothelial differentiation, characterized by cytokeratin expression and positivity for endothelial markers such as CD31, ERG, and FLI1 (2, 4, 15). CD34 is usually negative in tumor cells or restricted to vascular structures, consistent with the immunophenotype observed in the present cases (2, 4, 15). The main differential diagnosis is epithelioid sarcoma, which may show similar epithelioid morphology and cytokeratin expression. However, endothelial marker expression and nuclear FOSB positivity favor PHE (2, 4, 6). Furthermore, retained INI1/SMARCB1 expression may provide additional diagnostic support, as loss of nuclear INI1/SMARCB1 expression is characteristic of epithelioid sarcoma (17). Other vascular tumors, particularly epithelioid hemangioendothelioma (EHE), should also be considered. In this context, the combined use of FOSB and CAMTA1 IHC may facilitate differential diagnosis, as PHE typically demonstrates nuclear FOSB expression, whereas classic EHE is more commonly associated with nuclear CAMTA1 expression (6, 18). In osseous lesions, osteolytic destruction, osteogenic changes, and cytokeratin expression may raise suspicion for osteosarcoma or metastatic carcinoma (1, 2, 15). Benign vascular lesions represent an important diagnostic consideration, particularly in Case 3, in which the well-defined, mildly T2-hyperintense lesions in the humerus and upper arm were initially interpreted as hemangiomas (8, 9). However, the regional and multifocal distribution of the lesions, involving the humeral medullary cavity, subcutaneous tissue, and skeletal muscle, together with gradual enlargement and the subsequent onset of pain, may raise suspicion for PHE (3, 9, 15). Nevertheless, these findings are not diagnostic, and histopathological confirmation remains necessary. Histologically, benign vascular lesions such as epithelioid hemangioma typically display a lobular vasoformative architecture with well-formed vascular lumina lined by relatively bland endothelial cells; immunohistochemically, they commonly express CD31 and CD34 (15). In contrast, the tumor in Case 3 was composed of spindle-shaped to epithelioid cells arranged in a solid, infiltrative growth pattern, with some cells exhibiting rhabdomyoblast-like morphology, features more consistent with PHE than with conventional benign vascular lesions. Although CD31 positivity supports endothelial differentiation, this finding alone is insufficient for definitive distinction. Moreover, FOSB expression may also occur in epithelioid hemangioma and should therefore not be interpreted in isolation. Therefore, the diagnosis should be based on the integration of histomorphology, the broader IHC profile, and the characteristic distribution pattern of the disease (6). In our cases, the combination of FOSB and INI1/SMARCB1 positivity, endothelial marker expression, CAMTA1 negativity, and absent or vascular-restricted CD34 expression supported the diagnosis of PHE. The key clinical, radiological, IHC, and molecular features that distinguish PHE from its principal mimics are summarized in Supplementary Table S1.

For localized PHE, surgical resection remains the mainstay of treatment, particularly for symptomatic, progressive, or functionally compromising lesions (3, 4, 16). In cases with osseous involvement, treatment strategies should be individualized according to tumor location, disease extent, structural stability, and recurrence risk, as limited resection alone may be insufficient when lesions involve weight-bearing bones or anatomically complex sites (15, 16). In the present case series, all three patients underwent surgical treatment because of spinal canal stenosis with spinal cord compression, suspected femoral recurrence with extensive bone destruction, or combined humeral and soft tissue involvement. Previous reports have suggested that systemic or adjuvant therapies, including sirolimus, denosumab, pamidronate, and other targeted agents, may be considered for multifocal, recurrent, unresectable, or bone-predominant PHE; however, the available evidence remains limited, and standardized treatment protocols have not yet been established (16, 19, 20). Therefore, treatment decisions should be guided by multidisciplinary evaluation, with careful consideration of imaging findings, pathological confirmation, surgical feasibility, functional preservation, and patient preference. Given the high risk of local recurrence in PHE, long-term postoperative surveillance, including clinical assessment and appropriate imaging follow-up, is essential, particularly in patients with osseous involvement, recurrent disease, or in those with pulmonary nodules suspicious for metastasis (13, 16).

Although PHE usually follows an indolent clinical course, recurrence remains an important clinical concern. Previous clinical studies have shown that PHE has a propensity for local recurrence, particularly in patients with multifocal disease, deep soft tissue involvement, or inadequate local control (3, 4, 16). This recurrence tendency may be related to the characteristic multiplanar growth pattern of PHE, in which lesions can involve the skin, subcutaneous tissue, skeletal muscle, and bone either synchronously or sequentially, making complete tumor clearance challenging (3, 5, 15). In the second case of the present series, the patient had previously undergone femoral tumor resection at another institution; recurrent disease subsequently developed in the proximal femur, followed by another recurrence in the thigh soft tissue 8 months after surgery. Although distant metastasis is uncommon, pulmonary metastases from PHE have been reported (4, 21). Therefore, the multiple pulmonary nodules observed in the second case should be interpreted cautiously and require close imaging surveillance, particularly given the patient's history of repeated recurrence. These findings suggest that follow-up for PHE should not be confined to the primary surgical site but should also include assessment for multifocal recurrence and possible distant disease, especially in patients with osseous involvement, recurrent tumors, or suspicious pulmonary findings (3, 4, 16, 21).

A limitation of this case series is the absence of molecular testing for FOSB rearrangements or specific fusion transcripts. Although the characteristic histomorphological and IHC findings, including nuclear FOSB expression and positivity for vascular endothelial markers, support the diagnosis of PHE, molecular testing could provide additional diagnostic support, particularly for tumors arising at unusual anatomical sites or in patients with atypical demographic characteristics. In future similar cases, molecular techniques such as FISH, RT-PCR, or RNA sequencing should be considered when sufficient tissue and appropriate technical resources are available.

In conclusion, we report three cases of PHE involving atypical skeletal sites, including the thoracic spine, femur, and humerus, with one case showing associated soft tissue involvement in the upper arm. Multimodal imaging, including CT, MRI, and PET/CT, was used to define lesion extent, assess skeletal and soft tissue involvement, and guide surgical planning. Surgical management was individualized according to tumor location, disease extent, and functional considerations. The final diagnosis was established by histopathological and IHC evaluation, particularly nuclear FOSB positivity and endothelial marker expression. Notably, one patient developed postoperative local recurrence and pulmonary nodules suspicious for metastasis. Although metastatic disease was not histologically confirmed, these findings warrant further evaluation and close surveillance. These cases emphasize that PHE should be considered in the differential diagnosis of atypical bone and soft tissue lesions, even in patients outside the typical demographic profile or in uncommon anatomic locations. Comprehensive imaging assessment, pathological confirmation, individualized treatment planning, and long-term surveillance are essential for improving diagnostic accuracy and clinical management.

## Data Availability

The raw data supporting the conclusions of this article will be made available by the authors, without undue reservation.

## Supplementary material

Supplementary Figure S1 **Histopathological and IHC features of thoracic vertebral PHE. (A)** Tumor cells infiltrating the surrounding adipose tissue. **(B)** Focal tumor necrosis was observed. **(C)** Collagen deposition was present within the tumor stroma. **(D)** Foci of trabecular bone formation and hemosiderin deposition were identified. **(E)** The tumor was composed predominantly of short spindle-shaped cells arranged in sheets. **(F)** Some tumor cells showed abundant eosinophilic cytoplasm and eccentrically located nuclei, resembling rhabdomyoblast-like cells. **(G)** CK-PAN was positive in tumor cell. **(H)** CD31 was positive in tumor cell. **(I)** Nuclear FOSB positivity was observed in tumor cells. **(J)** ERG was positive in tumor cells. **(K)** INI1/SMARCB1was positive in tumor cells. **(L)** CD34 staining was absent in tumor cells and mainly highlighted vascular structures. **(M)** CAMTA1 was negative in tumor cells. **(N)** Desmin was negative in tumor cells.

Supplementary Figure S2 **Histopathological and IHC features of femoral PHE. (A, B)** Tumor cells infiltrating adipose tissue, with focal trabecular bone formation and multinucleated giant cells. **(C)** Lymphocytic infiltration was observed within the tumor. **(D)** Foci of neutrophilic infiltration were present. **(E)** Some tumor cells showed abundant eosinophilic cytoplasm and eccentrically displaced nuclei. **(F)** Rhabdomyoblast-like tumor cells were observed. **(G)** CK-PAN was positive in tumor cell. **(H)** CD31 was positive in tumor cell. **(I)** FLI1 expression was observed in tumor cells. **(J)** ERG was positive in tumor cells. **(K)** Nuclear FOSB positivity was detected. **(L)** INI1/SMARCB1was positive in tumor cells. **(M)** CD34 staining mainly highlighted vascular structures and was negative in tumor cells. **(N)** CAMTA1 was negative in tumor cells.

Supplementary Figure S3 **Histopathological findings of recurrent PHE. (A, B)** Epithelioid and spindle tumor cells with mild cytologic atypia.

Supplementary Figure S4 **Histopathological and IHC features of humeral and soft tissue PHE. (A)** Tumor infiltration into skeletal muscle. **(B)** Hemangioendothelioma-like slit-like vascular spaces were observed. **(C)** Foci of trabecular bone formation were present. **(D)** The tumor contained epithelioid tumor cells arranged in sheets. **(E)** Some tumor cells showed rhabdomyoblast-like morphology. **(F)** CK-PAN was positive in tumor cell. **(G)** Nuclear FOSB positivity was observed in tumor cells. **(H)** INI1/SMARCB1was positive in tumor cells. **(I)** CD31 expression supported endothelial differentiation. **(J)** CD34 staining mainly highlighted vascular structures and was negative in tumor cells. **(K)** Desmin was negative in tumor cells. **(L)** CAMTA1 was negative in tumor cells.
