# Supplementary material for: Case Report: Three rare cases of pseudomyogenic hemangioendothelioma with atypical skeletal involvement
Source: Front Med (Lausanne). 2026 Sep 29;13:1934676. doi: 10.3389/fmed.2026.1934676 (PMC13658082; doi:10.3389/fmed.2026.1934676)
Supplement: Supplementary file 1 [file Table3.docx]

**Supplementary Table S1. Differential diagnosis of PHE and its principal clinical, radiological, IHC, and molecular mimics**

| **Entity** | **Clinical** **features** | **Radiological** **features** | **IHC features** | | | | | | | | | | | | | | **Molecular and other** **distinguishing features** |
| --- | --- | --- | --- | --- | --- | --- | --- | --- | --- | --- | --- | --- | --- | --- | --- | --- | --- |
|  |  |  | **CK-PAN** | **CD31** | **CD34** | | **FOSB** | | **CAMTA1** | | **TFE3** | | **Desmin** | | **INI1/** **SMARCB1** | |  |
| **PHE** **[1, 2, 3-7, 8, 9]** | Young adults; male predominance; multifocal extremity bone and soft-tissue lesions; local recurrence. | Often multifocal osteolytic lesions; soft-tissue extension; T2 hyperintensity; variable enhancement/FDG uptake. | +;  focal or diffuse | +;  focal or diffuse | | - | | Strong, diffuse nuclear + | | - | | - | | - | | Usually retained | Recurrent SERPINE1::FOSB, ACTB::FOSB, WWTR1::FOSB, or CLTC::FOSB fusions; rhabdomyoblast-like morphology. |
| **ES** **[9, 10-12]** | Adolescents/young adults; distal extremities or proximal trunk or pelvis; recurrent, potentially aggressive nodules. | Infiltrative superficial or deep soft-tissue mass; necrosis; occasional secondary osseous invasion. | + | - | | +/-; frequent | | -; weak focal possible | | - | | - | | - | | Lost in ~90% | SMARCB1 deletion or inactivation and loss of INI1 nuclear expression. ERG/FLI1 expression alone does not exclude ES. |
| **EHE** **[9, 10, 13-15]** | Adults; bone, soft tissue, liver, or lung; may be multifocal; variable malignant potential. | Multifocal lytic bone lesions or enhancing visceral or soft-tissue nodules; infiltrative lesions may coexist. | +/-; often focal | + | | + | | -; weak focal possible | | Usually nuclear + | | Variable | | - | | Retained | WWTR1::CAMTA1 in most cases; YAP1::TFE3 in a smaller subset. |
| **EAS** **[10, 16, 17]** | Usually middle-aged or older adults; aggressive soft-tissue or visceral tumor; early recurrence or metastasis. | Destructive, infiltrative mass with hemorrhage, necrosis, heterogeneous enhancement, and possible bone invasion. | +/- | +; usually diffuse | | +/- | | -; weak focal possible | | -; weak focal possible | | - | | - | | Retained | No defining FOSB or CAMTA1 fusion; MYC amplification supports radiation-associated angiosarcoma. |
| **Osteosarcoma** **[18-20]** | Commonly adolescents or young adults; painful metaphyseal long-bone lesion, frequently around the knee. | Aggressive mixed lytic and sclerotic lesion; mineralized osteoid matrix; periosteal reaction; soft-tissue mass. | -; rare focal possible | - | | - | | Not typical | | - | | - | | - | | Usually retained | Malignant osteoid; SATB2 positivity is supportive but nonspecific. TP53/RB1 alterations; MDM2/CDK4 amplification in low-grade subsets. |

**Abbreviations:** PHE, pseudomyogenic hemangioendothelioma; IHC, immunohistochemical; ES, epithelioid sarcoma; EHE, epithelioid hemangioendothelioma; EAS, epithelioid angiosarcoma.

**Interpretation:** "+", usually positive; "-", usually negative; "+/-", variable or focal. The double colon (“::”) denotes a gene fusion; for example, SERPINE1::FOSB indicates a fusion between SERPINE1 and FOSB.

**References**

1. Hornick JL, Fletcher CD. Pseudomyogenic hemangioendothelioma: a distinctive, often multicentric tumor with indolent behavior. *Am J Surg Pathol*. 2011;35(2):190-201.
2. Hung YP, Fletcher CD, Hornick JL. FOSB is a Useful Diagnostic Marker for Pseudomyogenic Hemangioendothelioma. *Am J Surg Pathol*. 2017;41(5):596-606.
3. Amary MF, O'Donnell P, Berisha F, et al. Pseudomyogenic (epithelioid sarcoma-like) hemangioendothelioma: characterization of five cases. *Skeletal Radiol*. 2013;42(7):947-57.
4. Agaram NP, Zhang L, Cotzia P, Antonescu CR. Expanding the Spectrum of Genetic Alterations in Pseudomyogenic Hemangioendothelioma With Recurrent Novel ACTB-FOSB Gene Fusions. *Am J Surg Pathol*. 2018;42(12):1653-1661.
5. Walther C, Tayebwa J, Lilljebjörn H, et al. A novel SERPINE1-FOSB fusion gene results in transcriptional up-regulation of FOSB in pseudomyogenic haemangioendothelioma. *J Pathol*. 2014;232(5):534-40.
6. Panagopoulos I, Lobmaier I, Gorunova L, Heim S. Fusion of the genes WWTR1 and FOSB in pseudomyogenic hemangioendothelioma. *Cancer Genomics Proteomics*. 2019;16(4):293–298.
7. Bridge JA, Sumegi J, Royce T, Baker M, Linos K. A novel CLTC-FOSB gene fusion in pseudomyogenic hemangioendothelioma of bone. *Genes Chromosomes Cancer*. 2021;60(1):38-42.
8. Inyang A, Mertens F, Puls F, et al. Primary Pseudomyogenic Hemangioendothelioma of Bone. *Am J Surg Pathol*. 2016;40(5):587-98.
9. Sugita S, Hirano H, Kikuchi N, et al. Diagnostic utility of FOSB immunohistochemistry in pseudomyogenic hemangioendothelioma and its histological mimics. *Diagn Pathol*. 2016;11;11(1):75.
10. Hornick JL, Dal Cin P, Fletcher CD. Loss of INI1 expression is characteristic of both conventional and proximal-type epithelioid sarcoma. *Am J Surg Pathol*. 2009;33(4):542-50.
11. McCarville MB, Kao SC, Dao TV, et al. Magnetic resonance and computed tomography imaging features of epithelioid sarcoma in children and young adults with pathological and clinical correlation: a report from Children's Oncology Group study ARST0332. *Pediatr Radiol*. 2019;49(7):922-932.
12. Stockman DL, Hornick JL, Deavers MT, Lev DC, Lazar AJ, Wang WL. ERG and FLI1 protein expression in epithelioid sarcoma. *Mod Pathol*. 2014;27(4):496-501.
13. Doyle LA, Fletcher CD, Hornick JL. Nuclear Expression of CAMTA1 Distinguishes Epithelioid Hemangioendothelioma From Histologic Mimics. *Am J Surg Pathol*. 2016;40(1):94-102.
14. Flucke U, Vogels RJ, de Saint Aubain Somerhausen N, et al. Epithelioid Hemangioendothelioma: clinicopathologic, immunhistochemical, and molecular genetic analysis of 39 cases. *Diagn Pathol*. 2014;1;9:131.
15. Antonescu CR, Le Loarer F, Mosquera JM, et al. Novel YAP1-TFE3 fusion defines a distinct subset of epithelioid hemangioendothelioma. *Genes Chromosomes Cancer*. 2013;52(8):775-84.
16. Wu J, Li X, Liu X. Epithelioid angiosarcoma: a clinicopathological study of 16 Chinese cases. *Int J Clin Exp Pathol*. 2015;8(4):3901-9.
17. Guo T, Zhang L, Chang NE, Singer S, Maki RG, Antonescu CR. Consistent MYC and FLT4 gene amplification in radiation-induced angiosarcoma but not in other radiation-associated atypical vascular lesions. *Genes Chromosomes Cancer*. 2011;50(1):25-33.
18. Conner JR, Hornick JL. SATB2 is a novel marker of osteoblastic differentiation in bone and soft tissue tumours. *Histopathology*. 2013;63(1):36-49.
19. Dujardin F, Binh MB, Bouvier C, et al. MDM2 and CDK4 immunohistochemistry is a valuable tool in the differential diagnosis of low-grade osteosarcomas and other primary fibro-osseous lesions of the bone. *Mod Pathol*. 2011;24(5):624-37.
20. Chen X, Bahrami A, Pappo A, et al. Recurrent somatic structural variations contribute to tumorigenesis in pediatric osteosarcoma. *Cell Rep*. 2014;10;7(1):104-12.
